# Prolonged P-Wave and QT Dispersion in Children with Inflammatory Bowel Disease in Remission

**DOI:** 10.1155/2017/6960810

**Published:** 2017-02-21

**Authors:** Helen Aghdasi Bornaun, Nuh Yılmaz, Günsel Kutluk, Reyhan Dedeoğlu, Kazım Öztarhan, Gonca Keskindemirci, Aras Tulunoğlu, Fatih Şap

**Affiliations:** ^1^Department of Pediatric Cardiology, Kanuni Sultan Süleyman Education and Research Hospital, İstanbul, Turkey; ^2^Department of Pediatric Cardiology, Medical Faculty, Mustafa Kemal University, Hatay, Turkey; ^3^Department of Pediatric Gastroenterology, Kanuni Sultan Süleyman Education and Research Hospital, İstanbul, Turkey; ^4^Department of Pediatric Cardiology, Cerrahpaşa Medical Faculty, İstanbul University, İstanbul, Turkey; ^5^Department of Pediatrics, Kanuni Sultan Süleyman Education and Research Hospital, İstanbul, Turkey; ^6^Department of Pediatric Cardiology, Medical Faculty, Necmettin Erbakan University, Konya, Turkey

## Abstract

*Objectives.* Ulcerative colitis (UC) and Crohn's disease (CD) are chronic inflammatory bowel diseases (IBD) with unclear underlying aetiologies. Severe cardiac arrhythmias have been emphasised in a few studies on adult IBD patients. This study aimed to investigate the alteration of the P-wave and QT interval dispersion parameters to assess the risk of atrial conduction and ventricular repolarisation abnormalities in pediatric IBD patients.* Patients and Methods.* Thirty-six IBD patients in remission (UC: 20, CD: 16) aged 3–18 years and 36 age- and sex-matched control patients were enrolled in the study. Twelve-lead electrocardiograms were used to determine durations of P-wave, QT, and corrected QT (QTc) interval dispersion. Transthoracic echocardiograms and 24-hour rhythm Holter recordings were obtained for both groups.* Results.* The P-wave dispersion, QT dispersion, and QTc interval dispersion (Pdisp, QTdisp, and QTcdisp) were significantly longer in the patient group. The mean values of Pminimum, Pmaximum, and QTcminimum were significantly different between the two groups. The echocardiography and Holter monitoring results were not significantly different between the groups. Furthermore, no differences in these parameters were detected between the CD and UC groups.* Conclusion.* Results suggest that paediatric IBD patients may carry potential risks for serious atrial and ventricular arrhythmias over time even during remission.

## 1. Introduction

Inflammatory bowel disease (IBD), which has two principle types of ulcerative colitis (UC) and Crohn's disease (CD), is composed of gastrointestinal inflammatory conditions [1, 2]. Genetic susceptibility and environmental factors are accused at the etiology of the diseases [1, 2]. Multiple extraintestinal manifestations are observed in approximately 23%–42% of IBD patients [1, 2]. Among the extraintestinal complications, cardiac involvement represents one of the leading causes of morbidity and mortality [1–3]. There are evidences which suggested that IBD patients are at higher risk of cardiovascular disease (CVD) [3]. Cardiac involvement has been reported extensively in IBD patients such as recurrent perimyocarditis, valvular damage, venous thrombosis, and variety of cardiac arrhythmias [1, 2].

The autonomic nervous system has been known to be a major regulator system of the gastrointestinal physiological functions [4, 5]. According to the traditional view of the pathogenesis of IBD, there is uncontrolled inflammation in the gastrointestinal system which is mediated with proinflammatory factors such as tumor necrosis factor-*α* (TNF-*α*), IL-1, and IL-6 [5, 6]. In particular, parasympathetic nervous system is sensitive to the proinflammatory cytokines [4, 6]. In the literatures, there are studies about arrhythmia risks in the chronic inflammatory disease such as rheumatoid arthritis and ankylosing spondylitis [6, 7]. Autonomic dysfunction may be one of the leading factors in the pathogenesis of cardiac involvement [1–3, 5].

Measurement of P and QT parameters reflects the autonomic function electrophysiologically [7–9]. Measurements of these parameters were an attempt to distinguish homogeneity from inhomogeneity in the myocardium [8, 9]. The changes in these parameters are a phenomenon that predisposes patients to an abnormal electric current of the heart [3, 10]. Arrhythmias such as atrial fibrillation (AF), atrioventricular (AV) block, and ventricular arrhythmia have been reported in some adult IBD patients [2, 11, 12]. Considering that cardiac involvement has poorer prognosis, it is mandatory to identify IBD patients at a high risk of CV problems for defining long-term management of the illness. On the other hand, the available data regarding the prevalence and mechanism of cardiac rhythm disturbances are limited and the impact of chronic inflammation on sinoatrial and AV conduction systems is not sufficiently clear in IBD patients [13].

There are a few publications concerning cardiac rhythm disturbances in adult IBD patients but there is only one letter to the editor in children with IBD [14]. Therefore, in the present study, we investigated the prevalence of electrocardiographic differences by measuring P dispersion, QT dispersion, and corrected QT dispersion (Pdisp, QTdisp, and QTcdisp) and their components in a cohort of pediatric IBD patients in their remission period. IBD generally affects young patients. The aim of this study is to detect asymptomatic cardiovascular disease in terms of arrhythmia in order to reduce the incidence of cardiovascular events in the future.

## 2. Materials and Methods

### 2.1. Patients and Controls

Thirty-six children who were diagnosed with IBD (20 with UC and 16 with CD) were prospectively enrolled in this study, when they were in remission phase. The local ethical committee of Istanbul Health Sciences University, Kanuni Sultan Süleyman Education and Research Hospital, approved the study, and an informed consent from parents and subjects was obtained.

The diagnosis of UC or CD was made on the basis of the combination of history, physical, and laboratory examinations and endoscopic, histopathological, and radiological findings of the upper and lower gastrointestinal system. These patients were followed up in the Pediatric Gastroenterology Department of our hospital for at least 12 months and they were all in remission phase. All patients were using mesalamine (50 mg/kg/day) plus azathioprine (2–2.5 mg/kg/day) but two patients with Crohn's disease, one female with perianally penetrating disease and one male with drug resistance, were on Infliximab (IFX) therapy. Disease activity of the subjects was evaluated according to the Pediatric Ulcerative Colitis Activity Index (PUCAI) [15] or Pediatric Crohn's Disease Activity Index (PCDAI) [16] scores, and C-reactive protein level (CRP), erythrocyte sedimentation rate (ESR), white blood cells (WBC) count, and platelet (PLT) count were investigated. No relapses of IBD were recorded during past 3 months before the Holter ECG tests were conducted. Patients with evidence of coexisting cardiac disease, using arrhythmogenic medication and having another systemic disease, acute or chronic infection, anaemia, electrolyte imbalances, or other diseases known to affect ECG, were excluded from the study.

The control group comprised 36 age- and sex-matched healthy children. All the healthy subjects were free of any medications. All patients underwent a detailed evaluation by the same pediatric cardiologist; the evaluation included medical history, physical examination, 12-lead ECG, echocardiography, and 24-hour Holter recordings (CardioNavigator Plus Impresario, Delmar Reynolds, Paris, France).

### 2.2. Echocardiographic Study

All the study subjects underwent a detailed echocardiography, which included valvular function evaluation and M-mode, two-dimensional, and colour examinations. Images were obtained on a General Electric S7 Pro (Florida, USA) equipped with a 3 MHz transducer. Standard transthoracic echocardiogram involving M-mode tracings was obtained at the level of tips of mitral leaflets in the parasternal long-axis position, and measurements of left ventricular end systolic dimension, left ventricular end diastolic dimension, and left ventricular ejection fraction were performed according to modified Simpson's method [17]. Moreover, left atrial and aortic root dimensions were measured using parasternal long-axis window in M-mode echocardiography.

### 2.3. Electrocardiography Analysis

A standard 12-lead ECG was obtained at a paper speed of 25 mm/s and an amplitude of 10 mm/mV (Nihon Kohden ECG 6511, Tokyo, Japan). The ECGs obtained from the study groups were uploaded to a computer using a high-resolution optical scanner. Therefore, we performed on-screen manual measurements on the computer. ECGs were studied with 0.02 ms mistake for heart rate and minimum (min) and maximum (max) wave durations and dispersion (Pmin, Pmax, Pdisp, QTmin, QTmax, QTdisp, QTcmin, QTcmax, and QTcdisp). Parameters were digitally measured on a standard 12-lead ECG. In this analysis, only the cycles with normal morphologic characterised beats were used; artefacts and ectopic complexes were excluded. All the patients had sinus rhythm in ECG measurement. Conduction time obtained from all of the 12 derivations was calculated.

The onset of P-wave was defined as the first elevation and depression from the isoelectric line on positive and negative waves, respectively [9, 17]. The point of return to the isoelectric line was defined as the end of the P-wave [9, 17]. Pmax and Pmin durations were measured for all patients. The difference between Pmax and Pmin durations on the ECG was defined as Pdisp [9, 17].

The QT interval was measured from the onset of the QRS complex to the end of the T-wave in each ECG lead [9, 17]. Biphasic T-waves were measured to the time of the final return to the baseline. If U-waves were present, the QT interval was measured to the base point of the curve between T-waves and U-waves [9, 17]. Leads with unclear T-waves were excluded. The longest (QTmax) and the shortest (QTmin) QT intervals were measured for all patients [9, 17]. The QT interval should have been measurable in at least eight derivations and in at least three QT intervals in a derivation [9, 17]. Extrasystolic and post-extrasystolic cycles were ignored. QTdisp was defined as the difference between QTmax and QTmin on a standard 12-lead ECG [9]. QTc intervals were calculated using Bazett's formula (QTc = QT interval/√R-R interval) [17]. The durations of the P-wave, QT, and QTc intervals and dispersion values were compared between the patient and control groups.

### 2.4. Analysis of 24-Hour Holter Monitoring

In this analysis, a standard ambulatory ECG recording system (Century 2000/3000 HRV Package System, version 1.32, Biomedical Systems, St. Louis, Missouri, USA) was used. All the recordings were evaluated by the same specialist. Supraventricular and ventricular dysrhythmic patterns determined by 24-hour recording were evaluated according to the Lown-Wolf classification [18]. The results of Holter ECG monitoring were compared between the two groups.

### 2.5. Statistical Analyses

All data were processed using the IBM SPSS statistical package, version 21.0 (SPSS Inc., Chicago, Illinois, USA). The normally distributed continuous variables were assessed using Student's *t*-tests to compare means for normally distributed data. Results are presented as mean ± standard deviation. Two or more standard deviations from the normal range were considered abnormal. A *P* value of less than 0.05 was considered statistically significant.

## 3. Results

The mean ages of patients and controls were 11.96 ± 4.47 and 12.01 ± 4.2 years, respectively, with 52.7% (*n* = 19) being females and 47.3% (*n* = 17) being males in each group. There was no significant difference between the mean systolic blood pressure values in the study and control groups (101.81 ± 10.37 and 98.41 ± 12.57 mmHg, resp.). There were also no significant differences in age, sex, and body mass index (BMI) between the groups. The basic demographic characteristics of the study and control groups are presented in Table 1.

The patients were all in remission phase (PUCAI score = 11.85 ± 5.16 and PCDAI score = 4.25 ± 2.3) and their CRP, ESR, and WBC and PLT counts were in normal range (Table 2). All patients were free of steroids and using mesalamine plus azathioprine; only two patients were on Infliximab therapy. The duration of the disease did not differ between CD and UC patients.

The 24-hour Holter ECG monitoring results showed normal findings in both groups. We observed a maximum of four extrasystoles in eight pediatric IBD patients during monitoring. A few children in the healthy group also had extrasystoles but less frequently; this difference was not statistically significant.

The results of echocardiography showed that the left ventricular end diastolic, left atrium, and aortic root dimensions of IBD patients were not significantly different from those of the control patients (*P* > 0.05) (Table 1). Mild degrees of mitral regurgitation were observed in nine (25%) patients and in seven (19%) controls (*P* > 0.05).

Although the minimum, maximum, and mean heart rates were higher in the IBD patients, the differences in these parameters between the two groups were not statistically significant (*P* > 0.05). Pmax was significantly higher and Pmin was significantly lower in the IBD group than in the control group (*P* = 0.009 and *P* = 0.002, resp.). With respect to QTmax, QTmin, QTcmax, and QTcmin, QTcmin was significantly lower in the IBD patients than in the control group (*P* = 0.02), but the other QT interval parameters were not significantly different between the groups (*P* > 0.05). With respect to the dispersion indexes, significantly higher differences were found in Pdisp, QTdisp, and QTcdisp in the IBD group than in the control group (*P* = 0.000) (Table 3).

In addition, the results were compared between pediatric CD and UC patients. The P-wave and QT interval parameters demonstrated no differences between the two groups (*P* > 0.05) (Table 4). Furthermore, the clinical, echocardiographic, and Holter ECG findings did not show any significant differences between the UC and CD groups.

## 4. Discussion

Cardiovascular complications are believed to be increased in IBD patients; this can worsen the prognosis of the disease [1, 2, 12]. To date, although the pathogenic mechanism underlying the secondary cardiac events is poorly understood, related studies have indicated that inflammatory mechanisms have an important role in the aetiology of IBD and perpetuation of arrhythmia [11, 12].

The inflammatory processes may affect endothelial damage/dysfunction, which may be preceded by abnormal depolarisation/repolarisation of the cardiac myofibrils [5, 6]. Conduction abnormalities leading to increased P-wave dispersion and QTc dispersion are detectable on surface ECG [12, 18]. Increasing evidence indicates that systemic inflammation as measured by CRP is correlated with increased Pdisp in different diseases [19, 20]. Our patients were using anti-inflammatory drugs and, according to clinical and laboratory parameters, they were all in remission. However, because of the lack of laboratory data about proinflammatory cytokines, we could not prove that systemic inflammation was absolutely inhibited in these patients.

IBD drugs with possible arrhythmogenic potential include mesalamine, azathioprine, corticosteroids, and biological drugs such as IFX [14, 21–24]. There are no significant documented pediatric data relating to QT alteration or fatal dysrhythmias with these drugs. About arrhythmogenic side effects of mesalamine and azathioprine, only few case reports in adult IBD patients are available in the literatures [21, 22]; however, a significant number of studies suggest that biologic drugs may increase the risk of arrhythmia in adult patients with inflammatory conditions [14, 23, 24]. In pediatric population, there is only one letter to the editor which is discussing cardiac involvement in 7 of 12 children with IBD during IFX therapy. However, because of lack of cardiovascular screening before the treatment, they could not exclude the presence of heart disease before IFX treatment [14]. Nuti et al. [24] evaluated the long-term efficacy and safety of biological therapy in 78 pediatric patients with CD followed up at a referral center and they did not report any cardiovascular side effects with these drugs. Except two patients who were receiving IFX, all of our patients were using mesalamine and azathioprine and their echocardiographic and Holter ECG findings did not show any significant differences between these different treatment modalities.

The parameters of P-wave and QT interval indices are influenced by age, sex, and BMI [25]; therefore, we selected age- and sex-matched control according to these parameters for comparison with the patient group. In this study, IBD patients showed a significantly higher duration of Pmax and Pdisp and a significantly lower duration of Pmin than individuals in the control group. The mean value of Pdisp in our study group was in upper normal limit (Pdisp = 39.22). As we have known, Pdisp value of >40 ms has been accepted as a marker for the risk of atrial arrhythmias [26]. This level close to risk limits may carry a potential risk of atrial arrhythmias in the future. Similar to our study, Dogan et al. [27] reported significant increases in Pdisp in 69 IBD patients.

Various studies have demonstrated that increased QTdisp may increase the occurrence of serious ventricular arrhythmias and even sudden death in many cardiac and noncardiac diseases [8, 17], although there are few trials that deny the prognostic value of QTdisp [27]. A study by Coruzzi et al. [11] demonstrated that changes in autonomic enteric regulation in IBD patients may also affect neural CV control even in the quiescent phase of illness; however, this is still a matter of debate. Similar autonomic imbalance may have a bearing on ventricular repolarisation abnormalities and further the pathogenesis of P-wave and QT interval prolongation in IBD patients. In the other study by Curione et al. [28], QTc interval and QTcdisp values were both considerably prolonged in IBD patients compared with individuals in the control group. The literatures suggested that sympathetic activity changes could be the reason for QT and QTc interval alterations and increased QT dispersion and QTc dispersion of ventricular repolarisation [29, 30]. In contrast to these studies, Dogan et al. did not find any significant difference in QTdisp values between 69 IBD patients and controls [27].

The results of our study had showed considerably increased dispersion of the QT and QTc parameters in pediatric IBD patients in comparison with controls, which was compatible with previous observations in adult IBD patients [31–33]. The values of QTdisp (44.33 ± 7.55 ms) were significantly higher than those in the control group (32.55 ± 6.77 ms). QTdisp of >40 ms is reported to have 88% sensitivity and 57% specificity for the development of ventricular repolarisation heterogeneity and is a marker for prediction of inducibility of sustained ventricular tachycardia during an electrophysiology study [34, 35]. In addition, our findings had showed a decrease in the minimum (QT and QTc) interval and prolongation of maximum (QT and QTc) intervals in the IBD group. However, only the difference in QTcmin was statistically significant. We think that these findings may possibly be related to an increase in sympathetic activity.

We noted that the heart rate of our patient group had a nonsignificant mild increase. This may represent a change in the cardiac autonomic response, which is known to be a factor impacting the changes of the QTc interval [32–34].

Although QTmax and QTcmax were not significantly prolonged, QTdisp and QTcdisp values were significantly higher in the patients than in the controls. These results may suggest that prolongation of QTdisp and its indices may reflect spatial differences in myocardial recovery times. This is the consequence of the combination of minimum and maximum of QT intervals rather than exclusive QT interval prolongation.

Our study did not reveal significant differences in clinical characteristics, presentation, ECG, Holter ECG, or echocardiographic findings between CD and UC patients. Therefore, UC and CD can show similar autonomic and ECG alternations owing to their same mechanisms of tissue damage [32].

Based on our results, despite ECG abnormalities, the absence of any clinically important arrhythmias on the 24-hour ECG monitoring of our patients may be owing to the fact that IBD is associated with a decreased sympathovagal modulation of the heart rate and with increased inhomogeneity of cardiac recovery times.

To the best of our knowledge, the current study is the first one designed to evaluate ECG abnormalities in pediatric IBD patients during remission. We speculate that these differences are secondary to the inflammatory condition of IBD and are not heart-related problems and the study may form the basis of the development of serious cardiac arrhythmias in the future.

## 5. Study Limitations

The relatively low number of patients and lack of long-term clinical follow-up are the major limitations of our study. Further large-scale, prospective, longitudinal studies are needed to elucidate the exact effect of electrocardiographic studies on the risk of serious dysrhythmias and sudden cardiac death in this special patient population.

## 6. Conclusion

The current study is the first paediatric report supporting the results of the few adult IBD studies. The effects of IBD on atrial conduction and ventricular repolarisation may cause the prolongation of the duration of P-wave and QT interval dispersion which occur earlier in life and may predispose patients to the development of serious atrial and ventricular arrhythmias in the long term. Therefore, it is advisable to perform frequent ECG examinations with accurate measurement parameters of the P-wave and QT variability in all paediatric IBD patients even if they have had no past CV diseases and are free of cardiac involvement. This may potentially result in an early diagnosis and treatment of at-risk children and adolescents, which would have clear benefits for their future health.

## Figures and Tables

**Table 1:** The demographic characteristics and echocardiographic features of the IBD and control group.

	Patients, CD (16) + UC (20)	Control (36)	*P*
Age (years)	12.37 ± 4.33	12.11 ± 4.49	NS
BMI (kg/m)	19.58 ± 3.98	18.41 ± 5.06	NS
Sex (male/female)	17 (47.2%)/19 (52.78%)	17 (47.22%)/19 (52.78%)	NS
Weight (kg)	44.19 ± 18.37	43.84 ± 18.2	NS
Height (cm)	146.80 ± 22.02	149.22 ± 23.45	NS
EF%	69.75 ± 5.72	70.64 ± 6.16	NS
LVEDD (cm)	43.47 ± 6.91	41.54 ± 6.65	NS
LA (cm)	2.63 ± 0.44	2.62 ± 0.35	NS
AO (cm)	2.29 ± 0.36	2.3 ± 0.34	NS

BMI: body mass index; LVEDD: left ventricle end diastolic diameter; EF: ejection fraction; LA: left atrium; AO: Aorta.

**Table 2:** Demographic, clinical, and laboratory features of the UC and CD groups.

	CD (16)	UC (20)	*P*
Age, years	12.8 ± 3.97	11.55 ± 4.8	NS
Weight (kg)	41.65 ± 17.65	146.01 ± 19.09	NS
Height (cm)	146.73 ± 23,15	146.86 ± 2.77	NS
Gender (males/females)	7/9	8/12	NS
Duration of IBD, months	16.5 ± 3.3	16.8 ± 3.6	NS
Extent of the disease at diagnosis	L1^*∗*^^ ^ *n* = 9	E2^#^ *n* = 2	
	L3^*∗∗*^^ ^ *n* = 6	E3^##^ *n* = 2	
	L3 B3p^*∗∗∗*^ *n* = 1	E4^###^ *n* = 16	

Values at cardiac examination			

WBC	6400 ± 1340 (10e3/*μ*L)	6950 ± 1120 (10e3/*μ*L)	NS
PLT	234000 ± 42000 (10e3/*μ*L)	215000 ± 39500 (10e3/*μ*L)	NS
CRP	3.2 ± 0.8 (mg/L)	2.9 ± 1.3 (mg/L)	NS
ESR	8 ± 4 (mm/H)	7 ± 6 (mm/H)	NS
Mean PCDAI score	4.25 ± 2.3		
Mean PUCAI score		11.85 ± 5.16	

WBC: white blood cells count; PLT: platelet count; CRP: C-reactive protein; ESR: erythrocyte sedimentation rate; PUCAI: Pediatric Ulcerative Colitis Activity Index; PCDAI: Pediatric Crohn's Disease Activity Index.

L1^*∗*^: terminal ileum; L2^*∗∗*^: colon; L3B3p^*∗∗∗*^: ileocolon perianally penetrating.

E2^#^: rectosigmoid + descending colon; E3^##^: rectosigmoid + descending + transverse colon; E4^###^: pancolitis.

**Table 3:** Comparison of demographic and electrocardiographic parameters between Ulcerative colitis (UC) and Crohn's disease (CD) groups.

	Patient	Control	*P*
HR/minute	91.44 ± 12.88	86.58 ± 11.38	0.094
HR (min)/minute	50.3 ± 87.05	47.38 ± 5.69	0.051
HR (max)/minute	166.19 ± 18.34	161 ± 18.34	0.169
P duration (min) ms	49.22 ± 8.27	53.33 ± 3.82	**0.009**
P duration (max) ms	88.44 ± 8.69	83.33 ± 4.4	**0.002**
P dispersion ms	39.22 ± 6.54	30 ± 6.54	**0.000**
QT (min), ms	298.88 ± 27.80	309.66 ± 21.3	0.069
QT (max), ms	343.22 ± 26.9	342.22 ± 21.34	0.861
QT dispersion ms	44.33 ± 7.55	32.55 ± 6.77	**0.000**
QTc (min), ms	352.77 ± 22.17	365.55 ± 22.17	**0.021**
QTc (max), ms	405.5 ± 23.56	404 ± 23.29	0.787
QTc dispersion ms	52.72 ± 10.61	38.44 ± 7.81	**0.000**

HR: heart rate; QTc: corrected QT; ms: millisecond.

**Table 4:** Comparison of electrocardiographic parameters between patients with inflammatory bowel disease and control group.

	CD	UC	*P*
HR/minute	91.87 ± 14.84	91.14 ± 11.66	0.87
HR (min)/minute	49.80 ± 7.62	50.81 ± 6.78	0.68
HR (max)/minute	169.20 ± 17.44	164.05 ± 19.08	0.41
P (min), ms	50.67 ± 9.64	48.19 ± 7.21	0.38
P (max), ms	89.07 ± 10.31	88.00 ± 7.59	0.72
P dispersion, ms	38.40 ± 7.06	39.81 ± 6.26	0.53
QT (min), ms	294.40 ± 21.42	302.10 ± 31.71	0.42
QT (max), ms	339.20 ± 18.09	346.10 ± 31.44	0.45
QT dispersion, ms	44.80 ± 6.79	44.00 ± 8.20	0,6
QTc (min), ms	348.67 ± 20.75	355.71 ± 23.19	0.36
QTc (max), ms	402.27 ± 26.62	407.81 ± 21.49	0.49
QTc dispersion, ms	53.60 ± 11.01	52.10 ± 10.56	0.68

HR: heart rate; QTc: corrected QT; ms: millisecond.
